# sIR: siRNA Information Resource, a web-based tool for siRNA sequence design and analysis and an open access siRNA database

**DOI:** 10.1186/1471-2105-8-178

**Published:** 2007-05-31

**Authors:** Jyoti K Shah, Harold R Garner, Michael A White, David S Shames, John D Minna

**Affiliations:** 1 Hamon Center for Therapeutic Oncology Research, University of Texas Southwestern Medical Center, Dallas, Texas 75390, USA; 2 Department of Internal Medicine, University of Texas Southwestern Medical Center, Dallas, Texas 75390, USA; 3 Eugene McDermott Center for Human Growth and Development, University of Texas Southwestern Medical Center, Dallas, Texas 75390, USA; 4 Center for Biomedical Inventions, University of Texas Southwestern Medical Center, Dallas, Texas 75390, USA; 5 Department of Biochemistry, University of Texas Southwestern Medical Center, Dallas, Texas 75390, USA; 6 Department of Pharmacology, University of Texas Southwestern Medical Center, Dallas, Texas 75390, USA; 7 Department of Cell Biology, University of Texas Southwestern Medical Center, Dallas, Texas 75390, USA

## Abstract

**Background:**

RNA interference has revolutionized our ability to study the effects of altering the expression of single genes in mammalian (and other) cells through targeted knockdown of gene expression. In this report we describe a web-based computational tool, siRNA Information Resource (sIR), which consists of a new open source database that contains validation information about published siRNA sequences and also provides a user-friendly interface to design and analyze siRNA sequences against a chosen target sequence.

**Results:**

The siRNA design tool described in this paper employs empirically determined rules derived from a meta-analysis of the published data; it uses a weighted scoring system that determines the optimal sequence within a target mRNA and thus aids in the rational selection of siRNA sequences. This scoring system shows a non-linear correlation with the knockdown efficiency of siRNAs. sIR provides a fast, customized BLAST output for all selected siRNA sequences against a variety of databases so that the user can verify the uniqueness of the design. We have pre-designed siRNAs for all the known human genes (24,502) in the Refseq database. These siRNAs were pre-BLASTed against the human Unigene database to estimate the target specificity and all results are available online.

**Conclusion:**

Although most of the rules for this scoring system were influenced by previously published rules, the weighted scoring system provides better flexibility in designing an appropriate siRNA when compared to the un-weighted scoring system. sIR is not only a comprehensive tool used to design siRNA sequences and lookup pre-designed siRNAs, but it is also a platform where researchers can share information on siRNA design and use.

## Background

Nobel laureates, Andrew Fire and Craig Mello discovered that the injection of double-stranded RNA (dsRNA) into the nematode C. elegans initiated a potent sequence-specific response which caused a robust interference with the gene expression of the gene containing the same sequence as the dsRNA. [1]. RNAi is mediated through dsRNA, in a process similar to post-transcriptional gene silencing (PTGS) in plants and quelling in fungi. PTGS is a gene regulatory process, where reduction in the steady-state levels of a specific mRNA occurs through sequence-specific degradation of the transcribed mRNA [2]. It is thought that this process evolved as a defense mechanism against RNA viruses. In organisms capable of RNAi, upon entry into the cytoplasm, long dsRNA is cleaved by an RNase III-like enzyme Dicer into small interfering RNA (siRNA) about 21–23 nucleotides in length. These siRNAs assemble into multiprotein RNA-inducing silencing complexes, which then bind to target mRNA using the antisense siRNA as a guide, cleaving the mRNA-siRNA complex. Higher metazoans have evolved different defense mechanisms against RNA viruses, and initiate the interferon response when dsRNA longer than 30 bps is detected in the cytoplasm. However, synthetic oligonucleotides 21–23 bps in length do mediate RNAi in these cells, without an interferon response [3].

RNAi technology has proven its usefulness in many fields including cancer, gene therapeutics, functional genomics, etc. [3-5]. It is currently the most popularly used gene-silencing technique in functional genomics [6]. Because of its wide range of applications and popularity; we sought to create a tool that can help design efficient siRNAs quickly. siRNA Information Resource ('sIR') is a web-based computational tool that aids in selecting the target sequence for siRNA within a specified target RNA. sIR provides an analysis platform which includes a weighted scoring system to predict siRNA efficacy as well as an open-source database that contains effectiveness information about siRNAs that have been published or tested in our laboratories. Considering the importance of the siRNA technology, we have pre-computed siRNAs for all the known human genes. This pre-computed database provides a list of siRNAs with highest possible score (greatest knockdown) and minimum number of BLAST hits with the Human Unigene database (greatest specificity).

## Implementation

sIR was built on a Linux based server. The databases involved were implemented locally to speed up the design process using a PostgreSQL database. PHP, Perl (CGI) and HTML scripts were written to communicate with the databases using a web based user interface. Other scripting languages such as Perl and Bioperl were used to implement the design algorithms as well as to parse BLAST output. MATLAB statistical software was used to analyze the siRNA data and to refine the weighted scoring algorithm. sIR uses a variety of information from genetic databases such as RefSeq, SOURCE, NCBI and similarity searching software such as BLAST to confirm the uniqueness of siRNA designs. Pre-computation analysis was performed on a Linux based cluster with 29 nodes. The siRNAs were computed in a parallel fashion using Sun Grid Engine 5.3 software for all the known human genes in the Refseq database.

## Results and discussion

**Figure 1:**
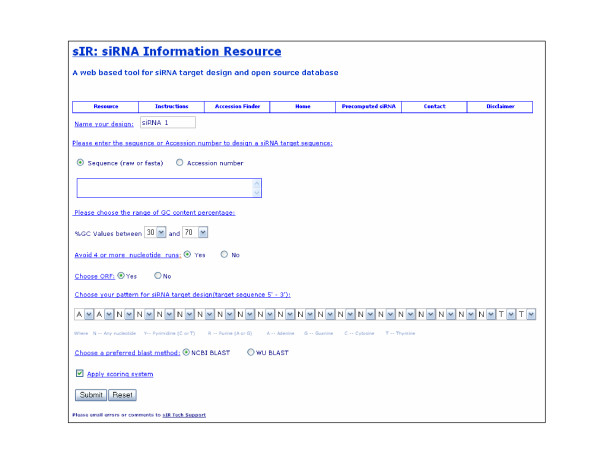
**Screen shot of siRNA information resource (sIR)**. sIR provides a menu interface which can be used to switch between various modes such as Target design mode, Resource (database) mode, Accession Finder mode and Pre-computed siRNA mode. The user can choose from among the siRNA pre-computed designs or input a sequence and select various input parameters such as sequence/accession number, percent GC content, maximal nucleotide runs, open reading frame, pattern and scoring system for a custom analysis.

The sIR implementation has four modes: the target design mode, the accession finder mode, the siRNA resource mode and the pre-computed siRNA database mode. The user can choose from parameters such as siRNA sequence pattern, GC content, maximal nucleotide runs and the application of a scoring system. Either a sequence (raw or FASTA format) or an accession number can be used as input. If neither is known, the software's "Accession Finder" mode can retrieve the accession numbers based on the gene aliases using a locally downloaded "SOURCE" database [7]. The outputs of the target design mode are target sequences. Figure 1 shows the screen shot of the sIR user interface.

### Target design algorithm

The target design algorithm uses various default and user-specified parameters to screen the mRNA sequence to identify and rank potential siRNA target sequences. If the Open Reading Frame (ORF) parameter is selected, the program pre-processes the input sequence by selecting an ORF 50 nucleotides before and after the 5' and 3' ends of the mRNA respectively. Then it searches for the target sequences with the user-specified pattern. If this pattern is not found then it looks for the following patterns in hierarchical order: AAN(19)TT, AAN(21), NAN(19)TT, NAN(21), where the character 'N' means any of the four bases and the number in the parenthesis after a nucleotide refers to the number of times that nucleotide repeats in the sequence [8]. For all the patterns found, the program assigns a score to the target sequence and displays the list of sequences in the descending order of the score. The user can choose one or more of these sequences to perform a customized similarity search using BLAST against a variety of databases such as Unigene (Human or Mouse), Refseq (Human or Mouse), Human Genomic or Ensemble cDNA transcripts. The user can choose a BLAST method, NCBI BLAST or WU BLAST and display results with a given similarity, e.g., similarity > 75% (16 bp/21 bp). NCBI BLAST uses a word size of 7 and is much faster than the more sensitive WU BLAST which uses a word size of 1.

Recent studies have shown that the overall identity search may not be able to detect all the off-target genes. According to Birmingham et al., (2006), off-targeting is associated with the presence of one or more 3' untranslated region (UTR) complementary matches with the hexamer or heptamer (position 2–7 or 2–8) to the antisense strand of the siRNA [9]. In this tool, users can further refine their alignment search by looking for exact matches of the seed region (position 12–18 of the 19 nt sense strand, complementary to position 2–8 of the antisense strand) in the 3' UTR mRNA regions from the human RefSeq database. The accession numbers with at least four seed matches in the 3' UTR mRNA are the listed in the descending order of the number of seed matches found.

### siRNA sequences

A database of ~1000 experimentally measured siRNA target sequences for ~400 different transcripts was populated as a training set for the system. These siRNA sequences were collected from various laboratories at the University of Texas Southwestern Medical Center, open source published databases and from published papers.

### Scoring system algorithm

A composite score is computed using rules we derived from various sources to rationalize the design process and to improve siRNA design success. The rules were compiled from various research papers [8,10-13]. For example, for the optimum design, it was found that the penultimate nucleotide of the antisense siRNA, which is complementary to position 2 of the 23nt target sequence, should always be complementary to the targeted sequence [8]. Primarily for simplification of chemical synthesis, TT is used. Hence the chance of having an efficient siRNA is increased if the position 2 of the target sequence is 'A'. In addition, moderately low GC content (30–55%) contributes to efficiency. Some studies have shown that the presence of elevated GC content at the 5' end of the siRNA target sequence improves the siRNA efficiency, whereas some studies have shown that there is no correlation [11,12]. Analyses based on the individual positions of the siRNA target sequence have shown that the presence of bases 'G/C' at position 1, 'A/U' at positions 15–19, 'A' at positions 3, 6 and 'U' at positions 10, 13 of the sense strands positively affect the siRNA efficiency, whereas the presence of bases 'G/C' at position 19, 'G' at position 13 and 'A/U' at position 1 negatively affect the siRNA efficiency [10-13].

Studies in the past as well as many recent studies have tried to find a correlation between secondary structure of sequences and potency of siRNA activity. Holen et al. (2002) have previously reported that there was no correlation between the M-Fold predicted secondary structures and siRNA efficiency [14]. However, some recent studies have shown that such a correlation may exist. Overhoff et al, (2005) have shown that the siRNA efficacy is related to the local RNA target structure and Poliseno et al, (2004) have proven that the energy profiling of siRNAs can be used to predict siRNA activity [15,16]. An attempt was made to test if the siRNA potency data correlated with the minimum free energy of the secondary structure of the siRNA. The m-fold server (version 3.2) was used to predict the minimum free energy values, dG, for all the 19 nucleotide siRNA sequences [17]. No correlation was found between the two. The relationship between secondary structures of sequences and siRNA potency is arguable and all the recent studies cannot be ignored. However, for simplification purposes, we have not considered this factor in our design of siRNA sequences.

In order to calculate the final score composed of the above mentioned parameters, it was important to introduce weights to rank the parameters in the order of their significance. The following fifteen criteria were tested and weights were calculated using the training dataset for each of them: I) A at position 2 of target siRNA sequence (21 nucleotides) II) A/U at position 1 of sense strand (19 nucleotides) III) G/C at position 1 of sense strand IV) A at position 6 of sense strand V) G at position 13 of sense strand VI)-X) A/U at positions 15–19 of sense strand XI) G/C at position 19 of sense strand XII) Moderate GC content XIII) A at position 3 of sense strand XIV) U at position 10 of sense strand XV) U at position 13 of sense strand [8,10-13].

The different weights of all the criteria were computed with the MATLAB 'Sum squared error' performance function to determine the best fit of the data using a second order polynomial fit. The best fit weights minimizing the sum squared error function were computed 200 times after randomizing the order in which the criteria were considered and the average weight for each criterion was computed. These were denoted as the final weights. After calculating appropriate weights it was realized that bases A at position 2 of target siRNA sequence, A at position 3 of sense and A/U at position 15 of the sense strand did not significantly affect the siRNA efficiency as they acquired the lowest weights. To reconfirm this observation, these 3 criteria were systematically removed from the scoring system calculations and the change in correlation between the score and efficiency was determined. There was no significant change so these 3 criteria were permanently removed from the scoring system. The weights were recalculated using the algorithm described above for the remaining twelve criteria and their weights were retained in the final scoring system. Table 1 shows the weights obtained for each criterion. Depending on these rules and weights, positive scores or penalties were assigned to a target siRNA sequence. The score of a siRNA sequence is defined by the following formula:

**Table 1:** Weights of the scoring system

**No**	**Criteria for the scoring system**	**Weight(W)**
A1	A/U at position 1 of sense strand	-1.4
A2	G/C at position 1 of sense strand	1.11
A3	A at position 6 of sense strand	0.70
A4	U at position 10 of sense strand	0.25
A5	G at position 13 of sense strand	-1.66
A6	U/T at position 13 of sense strand	0.31
A7	A/u at position 16 of sense strand	0.74
A8	A/U at position 17 of sense strand	1.20
A9	A/u at position 18 of sense strand	1.44
A10	A/U at position 19 of sense strand	0.87
A11	G/C at position 19 of sense strand	-1.02
A12	GC content	0.42

This table shows the different weights converged upon for the various parameters using a MATLAB algorithm. It can be observed that the criterion A5 with a base 'G' at position 13 of sense strand has the highest absolute weight whereas the criterion A4 with a base 'U' at position 10 of sense strand has the lowest absolute weight. The parameters with positive weights enhance the siRNA efficacy whereas the ones with negative weights suppress the efficacy of siRNA.

RawScoresiRNA=∑i=112WiAi
 MathType@MTEF@5@5@+=feaafiart1ev1aaatCvAUfKttLearuWrP9MDH5MBPbIqV92AaeXatLxBI9gBaebbnrfifHhDYfgasaacH8akY=wiFfYdH8Gipec8Eeeu0xXdbba9frFj0=OqFfea0dXdd9vqai=hGuQ8kuc9pgc9s8qqaq=dirpe0xb9q8qiLsFr0=vr0=vr0dc8meaabaqaciaacaGaaeqabaqabeGadaaakeaaieWacqWFsbGucqWFHbqycqWF3bWDcqWFtbWucqWFJbWycqWFVbWBcqWFYbGCcqWFLbqzdaWgaaWcbaGae83CamNae8xAaKMae8NuaiLae8Nta4Kae8xqaeeabeaakiabg2da9maaqahabaGaem4vaCLaemyAaKMaemyqaeKaemyAaKgaleaacqWGPbqAcqGH9aqpcqaIXaqmaeaacqaIXaqmcqaIYaGma0GaeyyeIuoaaaa@4AE8@

Where, *Wi *are the weights obtained by each of the twelve criteria (summarized in table 1) and *Ai *are the binary entries for each of them. They (*Ai*) take the value '1' if the criterion is satisfied and '0' if the criterion is not satisfied. The scores obtained were then normalized in the range of 0–100 using the following formula:

FinalScoresiRNA=(RawScoresiRNA−Scoremin⁡Scoremax⁡−Scoremin⁡)×100
 MathType@MTEF@5@5@+=feaafiart1ev1aaatCvAUfKttLearuWrP9MDH5MBPbIqV92AaeXatLxBI9gBaebbnrfifHhDYfgasaacH8akY=wiFfYdH8Gipec8Eeeu0xXdbba9frFj0=OqFfea0dXdd9vqai=hGuQ8kuc9pgc9s8qqaq=dirpe0xb9q8qiLsFr0=vr0=vr0dc8meaabaqaciaacaGaaeqabaqabeGadaaakeaaieWacqWFgbGrcqWFPbqAcqWFUbGBcqWFHbqycqWFSbaBcqWFtbWucqWFJbWycqWFVbWBcqWFYbGCcqWFLbqzdaWgaaWcbaGae83CamNae8xAaKMae8NuaiLae8Nta4Kae8xqaeeabeaakiabg2da9maabmaabaWaaSaaaeaajugybiabdkfasjabdggaHjabdEha3jabdofatjabdogaJjabd+gaVjabdkhaYjabdwgaLPWaaSbaaSqaaiabdohaZjabdMgaPjabdkfasjabd6eaojabdgeabbqabaGccqGHsisljugybiabdofatjabdogaJjabd+gaVjabdkhaYjabdwgaLPWaaSbaaSqaaiGbc2gaTjabcMgaPjabc6gaUbqabaaakeaajugybiabdofatjabdogaJjabd+gaVjabdkhaYjabdwgaLPWaaSbaaSqaaiGbc2gaTjabcggaHjabcIha4bqabaGccqGHsisljugybiabdofatjabdogaJjabd+gaVjabdkhaYjabdwgaLPWaaSbaaSqaaiGbc2gaTjabcMgaPjabc6gaUbqabaaaaaGccaGLOaGaayzkaaGaey41aqRaeGymaeJaeGimaaJaeGimaadaaa@7EAB@

Where Score_min _(= -4.08, sum of all the negative values in Table 1) and Score_max _(= 7.04, sum of all the positive values in Table 1) are the minimum and maximum possible score values respectively and FinalScore_siRNA _is the score obtained by the siRNA sequence after normalization of the raw score.

**Figure 2:**
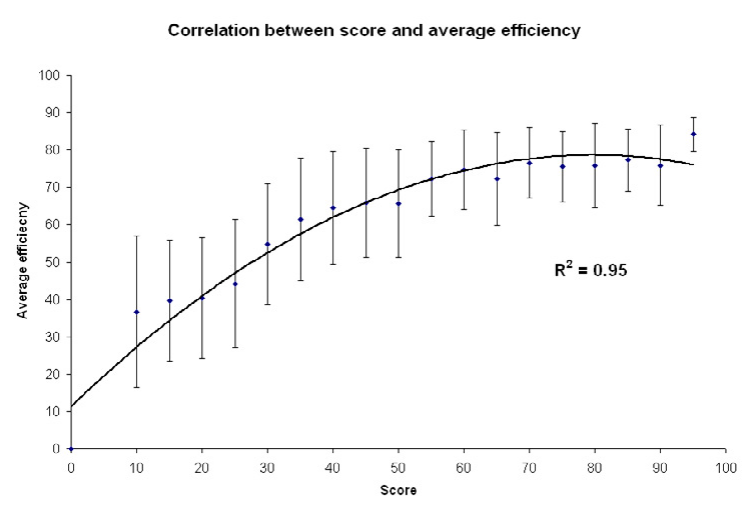
**Correlation between the score and average percentage efficiency**. The average efficiency was computed for a score bin size of 5. The error bars are the standard deviations in the average efficiency values. The data was fitted using a polynomial trend of second order and a correlation of 0.95 was observed.

The training set siRNA sequences were binned into 20 categories according to their scores and average efficiency value was calculated for each category. An excellent non-linear correlation of approximately 0.95 was observed between the score and percentage efficiency of the siRNA when a second order polynomial fit is used. This is depicted in Figure 2.

In order to test the validity of the siRNA design and the scoring system, two independent large datasets from previously published papers were evaluated. One test set comprised of the 500 most potent and the 500 least potent sequences out of 2,431 randomly selected siRNAs targeted to 34 reporter plasmids by Huesken et al, 2005 [18]. The average score of the most potent siRNAs was 63 and that of the least potent siRNAs was 38. To compare the effectiveness of this weighted scoring system, scores were computed for each of these 1000 siRNAs using three other popular design methods, "Rational design" (Reynolds et al), "Ui-Tei design" (Ui-Tei et al.) and "Amarzguioui design" (Amarzguioui et al.) respectively [10,12,13]. For rational design, the eight criteria mentioned in the paper were used for this purpose (maximum score: 10 and minimum score: -2) [10]. For Ui-Tei design and Amarzguioui design, the four criteria (maximum score: 4 and minimum score: 0) and six criteria (maximum score: 6 and minimum score: -5) rules mentioned in the respective papers were used [12,13]. In order to compare, the design scores were normalized using the same method as mentioned in equation II above. The scores were normalized such that the highest score corresponded to 100 and lowest to 0. After computing the normalized score using the three alternate design methods mentioned above, it was found that the average score of highly effective siRNAs with Rational was 50 and that of less effective siRNAs was 22. Similarly, the average score of highly effective siRNAs with Ui-Tei was 68, Amarzguioui was 63 and the average scores of less effective siRNAs with Ui-Tei was 40 and Amarzguioui was 41. Student T-tests (two-sample unequal variance) were performed on functional and non-functional siRNA scores to determine if the algorithms were able to distinguish the functional group from the non-functional group. All four algorithms were able to distinguish the two groups significantly and the sIR scoring system attained the lowest p-value. (Rational p-value = 2e-108, Ui-tei p-value = 3e-101, Amarzguioui p-value = 4e-91 and sIR p-value = 5e-115). The lower p-value shows that the means of the two groups (functional and non-functional) are significantly different. To evaluate further, linear correlation coefficients were computed between the reported activity of the siRNAs and scores of different algorithms. Again, sIR showed slightly higher correlation. (Rational R^2^-value = 0.4, Ui-tei R^2^-value = 0.33, Amarzguioui R^2^-value = 0.38 and sIR R^2^-value = 0.42). (See additional file 1: siRNA test set1). Similarly, we also tested siRNA sequences from a large repository of siRNA sequences, siRecords [19]. A total of 2579 unique siRNA sequences, independent of the sIR training set, from the April 2005 release of siRecords set were used. This dataset was divided into four categories according to the effectiveness of siRNAs, low, medium, high and very high. In order to test if the sIR algorithm is able to distinguish siRNAs between these four groups, one-way ANOVA was performed. The scores of the four groups were significantly different from each other with a p-value of 1.5e-18. The other three algorithms, Rational, Amarzguioui and Ui-tei were also able to significantly distinguish between the four groups with the p-values of 4e-12, 1.5e-18 and 1.8e-18 respectively. All these values are summarized in Table 2 below. (See additional file 2: siRNA test set2).

**Table 2:** Summary of test set scores

	**ANN**				**siRecords**				
	Least potent avg score	Most potent avg score	T-test p-value	Correlation between efficiency and Inhibitory activity	Low efficiency avg score	Medium efficiency avg score	High efficiency avg score	Very high efficiency avg score	One-way ANOVA test p-value

sIR	37.9	62.9	5.60E-115	0.422	50.7	57.9	58.3	60	1.51E-18
Amarzguioui	40.8	62.9	4.54E-91	0.34	51.2	58.1	59.1	60	1.51E-18
Ui-Tei	40.3	68.4	2.67E-101	0.39	52.6	60.9	61.8	64	1.84E-18
Rational	21.6	50.3	1.90E-108	0.4	34.7	39.5	40.2	43.8	4.03E-12

This table summarizes the scores attained by ANN (Huesken et al., 2005) and siRecords (Ren et al., 2006) sequences. siRNA scores were computed to compare weighted "sIR" and un-weighted "Rational design", "Ui-tei design" and "Amarzguioui design" scoring systems. This table shows the averages, p-values as well as correlation coefficient values obtained by the siRNA sequences in all four scoring systems.

Although all the algorithms performed fairly well with the two large data sets, sIR algorithm was consistently slightly better as it attained the highest correlation and the lowest p-values. Most of the rules for this scoring system were influenced by 'Tuschl rules' [8] and 'Rational design' [10] but the weighted scoring system provides better flexibility in designing an appropriate siRNA when compared to the un-weighted scoring systems.

### sIR Resource

The database or resource mode allows the researcher to search for existing and previously tested designs. The database includes the functional as well as non-functional siRNA sequences. There are several other repositories of siRNA sequences including some of the popular databases such as siRecords and HuSida [19,20]. HuSida currently consists of > 1100 siRNA sequence records and siRecords consists of > 4000 siRNA sequence records. HuSida was mainly designed to store functional human siRNA sequences with efficiency > 50%. However, it is of great research value to store the information of functional as well as non-functional siRNAs as it will help other investigators learn more about the nature of siRNA sequences. Both sIR resource as well as siRecords have the capability to store siRNA sequences from different species along with their variable efficacies. sIR resource also provides additional annotations such as miRNA seed region search to predict off-target activities along with sIR score. Some of the siRNA sequences are hyperlinked to images depicting their efficiency by Western Blot and other methods. This database can be updated using a password protected input form that accepts the data and images for new siRNA designs and uploads them to the database. Currently sIR database consists of only ~1200 siRNA sequences. It should also be noted that the data submitted in the sIR resource database is user-driven and hence there may be a user-related bias regarding the exact effectiveness of siRNA sequences. Hence users are encouraged to submit their research related data such as images of Western Blot analysis, plots etc. to be viewed by others. The sIR resource is envisioned to be a free, central repository where investigators can share their siRNA design and results.

### sIR pre-computed siRNA database

The growing popularity of siRNA calls for the need of a database which consists of pre-designed siRNAs for all the known human genes to provide simple and fast access to the designs. siRNAs were pre-computed for 24,502 genes in the human Refseq database. First, all the siRNAs were designed using the normal design parameters such as moderate GC content (30–55%), avoiding multiple nucleotide runs, using the open reading frame and score > 50. These siRNAs were evaluated for target specificity by performing a BLAST search against the Human Unigene database. siRNAs which had minimum number of blast hits (< = 2) were retained in the database. This enabled the selection of 3,907,079 siRNAs for 21,634 genes. The exact parameters used for the pre-computation of siRNA designs are listed in Table 3. The 95 genes which failed to produce siRNA designs using the above mentioned parameters were run again using less stringent parameters such as an expanded GC content limit (20–70%), allowing for multiple nucleotide runs, disregarding open reading frame and score > 40. Here again siRNAs with a minimum number of BLAST hits were retained in the database. This allowed us to select 1178 siRNAs for 73 of the 95 genes. On an average approximately 66 siRNAs per gene had scores > 80. The table containing a list of individual RefSeq accession numbers and the number of siRNAs designed for that gene with scores > 80 can be downloaded from the "Precomputed siRNA" tab of siRNA information resource website.

**Table 3:** Parameters used for the precomputation of siRNAs.

**Design parameters**	**Value**
Percent GC Content range	Moderate range, 30% to 50%
Multiple nucleotide run (Runs of 4 or more nucleotides)	Avoided
Open reading frame	An open reading frame region between 50 nucleotides from the 5' end and 50 nucleotides from the 3' end, downstream and upstream of the mRNA was considered for the design.
Score cutoff	Score > 50. siRNAs with scores > 50 were retained
Blast Hit cutoff	Number of blast hits < = 2. siRNAs with blast hits < = 2 and percent homology > 80% were retained.

This table lists the exact parameters used while pre-designing siRNAs for all the genes in the human RefSeq database.

In order to retrieve these pre-designed siRNAs the user can query the pre-computed siRNA mode with an accession number. The program returns the top 10 possible designs of the siRNAs for that particular accession number after sorting them with respect to minimum number of BLAST hits and then score. In an effort to avoid off-target effects, the user can choose to filter out siRNA sequences which have greater than three seed region matches within the 3' UTR mRNA sequences from the RefSeq database.

## Conclusion

sIR is not only a comprehensive tool used to design siRNA sequences and lookup pre-designed siRNAs, but it is also a platform where researchers can share information on siRNA design and use. It is difficult to find information about siRNA sequences which failed or had poor knockdown. It is however important to know that information, as it helps the researchers to avoid reinventing the wheel and enables computations like those herein. As of March 2007, the resource database consists of approximately 1200 entries comprising information on functional as well as non functional siRNAs which can be very important for future discoveries. This web based online tool along with the pre-computed siRNA database saves the investigator a lot of time.

Studies have shown that there is a relationship between siRNA sequence and the RNAi effect and that the presence of certain bases in a particular position contributes more to the efficiency of knockdown. The weighted scoring system of sIR was able to assign weights to different parameters which affect the siRNA potency. This validated system includes a suite of tools and databases that will allow researchers to rapidly and efficiently select siRNA designs with *a priori *specificity and efficacy estimates.

## Availability

• Project name: siRNA Information Resource (sIR)

• Project home page: 

## Authors' contributions

JKS developed the sequence processing algorithm, weighted scoring system and implemented the sIR software. JKS is currently responsible for its upkeep and upgrades. MAW and DSS contributed to the experimental validation of the program. HRG along with JDM outlined the project requirements, provided guidance and organizational support.

## Supplementary Material

Additional file 1 siRNA test set1. This file lists the 1000 siRNAs published in Huesken et al, 2005 (ANN, 500 most potent and 500 least potent) and their scores used for validation purpose. The scores for each of these siRNAs were computed using sIR algorithm, Rational design, Ui-Tei design and Amarzguioui design. Click here for file

Additional file 2 siRNA test set2. This file lists all the siRNAs from the siRecords database and their scores used for validation purpose. The scores for each of these siRNAs were computed using sIR algorithm, Rational design, Ui-Tei design and Amarzguioui design. Click here for file
